# Unveiling the antibacterial and antifungal potential of biosynthesized silver nanoparticles from *Chromolaena odorata* leaves

**DOI:** 10.1038/s41598-024-57972-5

**Published:** 2024-03-29

**Authors:** Ajit Kumar Bishoyi, Chita Ranjan Sahoo, Priyanka Samal, Nilima Priyadarsini Mishra, Bigyan Ranjan Jali, Mohd Shahnawaz Khan, Rabindra Nath Padhy

**Affiliations:** 1 Central Research Laboratory, Institute of Medical Sciences and Sum Hospital, Siksha ‘O’ Anusandhan Deemed to be University, Bhubaneswar, Odisha 751003 India; 2 Department of Clinical Hematology, Institute of Medical Sciences and Sum Hospital, Siksha ‘O’ Anusandhan Deemed to be University, Bhubaneswar, Odisha 751003 India; 3 Department of Biochemistry, IIT Ropar, Rupnagar, Punjab 140001 India; 4 Department of Chemistry, Veer Surendra Sai University of Technology, Burla, Sambalpur, Odisha 768018 India; 5 Department of Biochemistry, College of Science, King Saud University, 11451 Riyadh, Saudi Arabia

**Keywords:** Green synthesis, Silver nanoparticles, *Chromolaena odorata*, Antimicrobial activities, Molecular docking, Nanobiotechnology, Microbiology

## Abstract

This research investigates the biogenic synthesis of silver nanoparticles (AgNPs) using the leaf extract of *Chromolaena odorata* (Asteraceae) and their potential as antibacterial and antifungal agents. Characterization techniques like ultraviolet–visible, Fourier transform infrared (FTIR), Dynamic light scattering and zeta potential (DLS), X-ray diffraction (XRD), transmission electron microscopy (TEM), and field emission scanning electron microscopy and energy-dispersive X-ray spectroscopy (FESEM-EDX) confirmed the formation of spherical (AgNPs). UV–vis spectroscopy reaffirms AgNP formation with a peak at 429 nm. DLS and zeta potential measurements revealed an average size of 30.77 nm and a negative surface charge (− 0.532 mV). Further, XRD analysis established the crystalline structure of the AgNPs. Moreover, the TEM descriptions indicate that the AgNPs are spherical shapes, and their sizes ranged from 9 to 22 nm with an average length of 15.27 nm. The X-ray photoelectron spectroscopy (XPS) analysis validated the formation of metallic silver and elucidated the surface state composition of AgNPs. Biologically, *CO*-AgNPs showed moderate antibacterial activity but excellent antifungal activity against *Candida tropicalis* (MCC 1559) and *Trichophyton rubrum* (MCC 1598)*.* Low MIC values (0.195 and 0.390 mg/mL) respectively, suggest their potential as effective antifungal agents. This suggests potential applications in controlling fungal infections, which are often more challenging to treat than bacterial infections. Molecular docking results validated that bioactive compounds in *C. odorata* contribute to antifungal activity by interacting with its specific domain. Further research could pave the way for the development of novel and safe antifungal therapies based on biogenic nanoparticles.

## Introduction

Research on nanotechnology, a new and growing technology with many applications, will be at the forefront in the twenty-first century. To dramatically alter the physicochemical properties of these materials, new materials with diameters ranging from 1 to 100 nm are synthesized and used in various applications. The properties of nanoparticles can be influenced by their size, shape, and chemical makeup. Nanoparticles can be formed from a variety of materials^1,2^. Nanoparticles of silver, gold, platinum, and a few more metals are frequently used in medical applications^3–5^. Nanoparticles are used in several physical and chemical areas, including biological processes; the biosynthesis of nanoparticles is inexpensive and environmentally friendly. Bacteria, fungi, yeast, algae, and plant extracts are used to produce nanoparticles^6–8^.

To deal with nano-dimension in the growing field of nanotechnology, the traditional methods for creating nanoparticles require expensive equipment or high-end ingredients. However, the practices may not be environmentally safe^7,9^. So, “green” technologies are easy to use, convenient, eco-friendly, and economical for the synthesis of nanoparticles^10,11^. Green nanoparticle synthesis is a novel way to synthesize nanoparticles using biological sources like plants, algae, fungi, bacteria, yeast, etc.^12,13^. Due to its capacity for large-scale production at low cost and with minimal environmental impact, it is attracting attention. Although several noble nanoparticles have been employed for various uses, AgNPs have received the most attention due to their potential for use in the treatment and diagnosis of cancer^2,14^. Those nanomaterials are among the most essential and fascinating nanoparticles in biomedical applications. Their distinctive physicochemical features make them one of the most appealing nanomaterials in the field^15,16^.

The biosynthesized silver nanoparticles (AgNPs) have specific biological components, such as high scattering, tiny particle size, and a high surface area. The morphological structures of AgNPs have been reported to control several infections of wounds and burns, probably for the Ag component, and AgNPs have antibacterial, antifungal, anti-platelet, and antiviral activities^16,17^. Indeed, numerous commensal-bacterial species inhabit the human body, including the mouth cavity; bacteria and fungi cause the most common oral and skin-related infections^18,19^.

*Chromolaena odorata* (Family: Asteraceae), an indigenous plant from coastal Odisha with great diversity, was selected as the tested plant for the current study. Exploring their therapeutic potencies is the aim of the study (Fig. S1). This plant grows fast as a weed, is native to South and Central America, and is widely known as the Siam weed. *C. odorata* extract isolated from South Africa is reported for use in cuts, burns, soft tissue abnormalities, wound healing, anti-diarrheal, and to increase blood coagulation^20^. In the Southern part of Nigeria, leaves cure wounds, and skin ailments and prevent bleeding^21^. The chemical components of its phenolic chemical components have been identified as phytochemicals, alkaloids, tannins, flavonoids, and a few more phenolic compounds^22^. Due to the integument of mainstream medicinal chemistry principles, leading candidates derived from natural products have recently occupied a significant advantage in the pharmaceutical sector. Moreover, the Phyto-oil of *C. odorata* showed antibacterial activity against *B. cereus* and antifungal activity against *A. niger*^23^. Similarly, compounds from *C. odorata* identified, namely pectolinaringenin (1), ( ±)-4′,5,7-trimethoxy flavanone (2), 5-hydroxy-3,7,4′-trimethoxyflavone (3) and 3,5,7-trihydroxy-4′-methoxyflavone) (4) were isolated. Compounds 2 and 3 showed promising antimicrobial activity against bacteria *E. coli, S. aureus, and K. pneumoniae, as well as fungi A. fumigatus and C. neoformans, comparable to gentamicin, ciprofloxacin* and amphotericin B used as positive controls. Compounds 1 and 4 had shown good anti-biofilm and metabolic inhibition activities against *E. coli* and *S. aureus* but weak anti-adhesion activity^24,25^.

The fungal species *C. tropicalis* is a joint infectious agent in neutropenic patients and can spread to the skin. *T. rubrum* is a dermatophytes fungus colonizing the top layer of dead skin, causing ringworm, jock itching, and nail infection^26,27^*.*

This study used *C. odorata* aqueous extract to biosynthesize AgNPs (*CO*-AgNPs). Further, characterization was done by using ultraviolet–visible spectroscopy, Fourier transform infrared (FTIR), Dynamic light scattering, zeta potential (DLS and zeta potential), X-ray diffraction (XRD), transmission electron microscopy (TEM), and field emission scanning electron microscopy with energy-dispersive X-ray spectroscopy (FESEM-EDX). Moreover, biological activities were also performed against various strains including *S. aureus*, *S. pyogenes*, *E. coli*, *C. tropicalis*, and *T. rubrum* by Agar-well diffusion assay and minimum inhibitory concentration (MIC) assay. Furthermore, molecular docking was executed to examine the bioactive compounds of *Oscillatoria* sp. as ligands with bacterial and fungal proteins for their intermolecular binding analysis.

## Materials and methods

### Chemicals and plant sample collection

Silver nitrate (AgNO_3_, 169.87 g/moL) was purchased from Hi-media, Mumbai, India, and used without further treatment. *C. odorata* leaves were collected from the hospital campus in November, and the plant was identified and authenticated by the institutional plant taxonomist (Voucher number: PTBG0000025473). The leaves were cleaned and shade-dried for a week at room temperature. The dried leaves were crushed in a mortar and pestle to get powders. It was kept at 4 °C in an airtight container for further work as described^28^.

### Preparation of *C. odorata* leaf extract

The *C. odorata* leaf extract (50 mg/mL) was prepared in different solvents like acetone, ethanol, methanol, butanol, diethyl ether, n-hexane, and distilled water separately (Fig. S2) to ascertain the antibacterial activities of each solvent extract. However, the aqueous leaf extract was used for the biosynthesis of AgNPs. Briefly, 1gm powders were mixed/dissolved in 20 mL of double distilled water using a mortar and pestle. Next, the mixture was heated for about 15 min in a water bath at 40 °C, and the obtained material was collected after cooling and filtering with Whatman No. 1 filter paper^29^.

### Biosynthesis of silver nanoparticles

**Figure 1:**
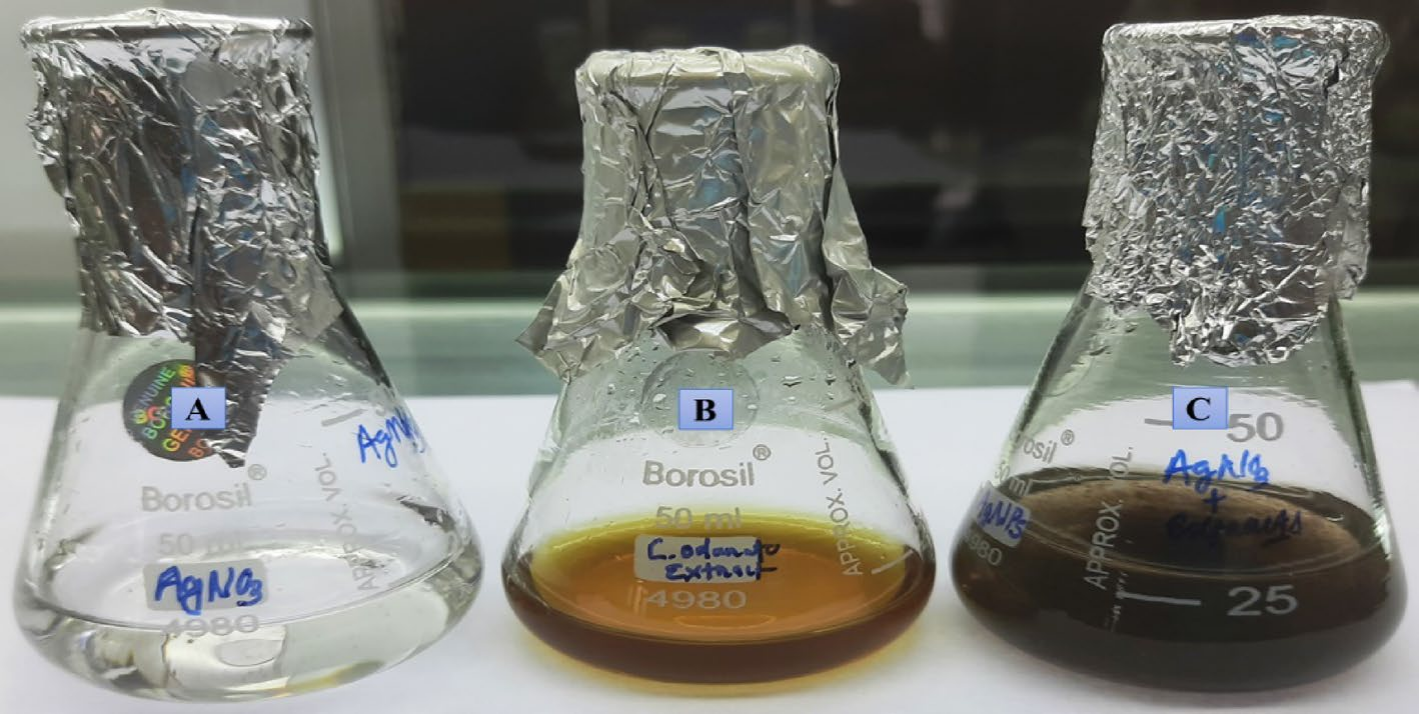
(**A**) The aqueous solution of 1 mM AgNO_3_. (**B**) *C. odorata* leaf extract. (**C**) Biosynthesis of *CO*-AgNPs from leaf extract of *C. odorata*.

An aliquot of 20 mL of aqueous leaf extract sample was added to a volume of 80 mL of 1 mM AgNO_3_ solution under magnetic stirring at 40 °C to get AgNPs. The reactive products turned from yellowish to dark brown after 15 min, indicating the formation of AgNPs (Fig. 1)^29^. After 1 h, the mixture was centrifuged for 20 min at 4000 rpm using a REMI R-8C centrifuge. The occurrence of AgNPs in the pellet was studied by scanning the sample with a Systronics spectrophotometer (Double beam spectrophotometer 2203) for UV–vis spectra between 300 and 750 nm wavelength^8,30^.

### Characterization

After UV–vis spectroscopy confirmation, the XRD patterns were analyzed using Rigaku Ultima IV X-ray diffraction. The power level was 40 kV with 40 mA, and the diffraction angle of two-theta (2Ө) was set between 5° and 60° to determine the size of crystals in powder. The crystallographic structure was determined by measuring X-ray intensities and scattering angles. The plane line width was used to determine the AgNPs from the XRD pattern^19,31, 32^.

The FTIR analysis of synthesized nanoparticles was conducted to determine the probable functional groups involved in nanoparticle capping and proficient stabilization. The *CO*-AgNPs were dried in a thermostatic desiccator at 45 °C for 24 h to avoid the presence of water molecules and thoroughly mixed with potassium bromide (5% of *CO*-AgNPs) and pressed to make thin discs. The spectrum of AgNPs from *C. odorata* leaf extract was recorded in the transmittance mode between 400 and 4000 cm^−1^ using JASCO FT/IR 4600-ATR spectrophotometer^16,33^.

Field emission scanning electron microscopy and energy-dispersive X-ray spectroscopy (Model: Carl Zeiss Supra 56) were also used to characterize AgNPs. To obtain a clear image of the cell surface, a drop of sample was poured onto a copper grid coated with carbon, and the excess was dried with blotting paper. The film on the FESEM grids was permitted to dry for 5 min by placing it under the mercury lamp to examine the material. The elemental composition of the synthesized sample was also analyzed using EDX spectroscopy. Before analysis, the synthesized sample was dried at room temperature. Moreover, the size and shape of the synthesized AgNPs were determined by transmission electron microscopy (TEM). The TEM descriptions of synthesized AgNPs were obtained using (FEI, Tecnai G2 TF30-ST)^34,35^.

DLS and zeta potential were measured for the size distribution of the small particles and molecules dissolved in a liquid medium. As a result, this measurement depends on factors such as particle size, surface distribution, particle concentration, and the type of ions present in the medium. The MALVERN instrument (ZEN 1600) was used to analyze the synthesized nanoparticles^33^.

To identify elements of the surface, X-ray photoelectron spectroscopy (XPS) studies were performed using multi-purpose X-ray photoelectron spectroscopy with a Sigma probe model. The deconvolution, curve fitting, and background subtraction were done using XPS Thermo Scientific™ Advantage software^34,35^.

### In-vitro antimicrobial studies

*The in-vitro* antimicrobial assessment was tested against pathogenic bacterial species *K. pneumoniae* (MTCC 1928), *S. aureus* (NCTC 6571), *E. coli* (NCTC 10418), and *S. pyogenes* (MTCC 1928), and fungal strains used were *C. tropicalis* (MCC 1559) and *T. rubrum* (MCC 1598). All these microorganisms were cultured/ maintained in the laboratory.

The antimicrobial activities of *CO*-AgNPs were monitored using the agar well diffusion method against *S. aureus, E. coli, S. pyogenes,* and *K. pneumoniae,* and the fungal strains *C. tropicalis* and *T. rubrum.* Briefly, With the help of a sterilized cork borer, wells of approximately 7 mm in diameter were cut on Muller-Hinton Agar (MHA) plates for bacteria and Sabouraud Dextrose Agar (SDA) plates for fungi. For the antimicrobial study, antibiotics Gentamicin 10 µg/mL and Nystatin 50 µg/mL were the positive controls for bacterial and fungal strains, respectively. The agar well diffusion plates were cultured for 18 h for bacteria and 48 h for fungi at 37 °C with 1 mg/ml of *CO*-AgNPs, and the plant powder extract was mixed with the chemical solvents^30,36^. The zones of inhibition (ZOI) were measured by measuring scale.

### Minimum inhibitory concentration (MIC)

The MIC was determined using a 96-well microplate as the concentration that inhibits bacterial/fungi growth. The corresponding wells were filled with the first 100 µL of the Sabouraud Dextrose Broth (SDB) for fungus and the Muller-Hinton broth (MHB) for bacteria plates. Then, aliquots of 1 mg/mL of *CO*-AgNPs prepared in sterilized distilled water were added in separate serial dilutions (No. 1–11) wells, with the last well acting as the control. The bacterial and fungal cultures were poured into 20 µL aliquots, and the 5 µL of 5% Triphenyl tetrazolium chloride (TTC) was placed into each well^30,37^. The bacterial and fungal plates were incubated at 37 °C in a BOD incubator for 18 h for bacteria and 48 h for fungi growth.

### Molecular docking

Target-suited molecules and proteins could be used to develop potent drug-able candidates in the docking process. Indeed, *C. odorata* had been reported with various bioactive compounds^23,24^, the ten bioactive compounds chosen were drawn on the ACD/ChemSketch software and were fully geometrically optimized using the open-label program^32^. Furthermore, the crystal structure of the bacterial protein dihydrofolate synthetase protein data bank (PDB) ID: 1AD1, 1AJ0 (2.3 Å) was retrieved from the PDB; as the crystal structure of the fungal protein Lanosterol C14 -Demethylase PDB ID: 5V5Z was used for docking purposes^30^. Furthermore, the molecular modeling of selected bioactive compounds with gram-positive (*S. aureus*) and gram-negative (*E. coli*) bacterial protein, dihydrofolate synthetase, was executed by Auto Dock software v4.2.6^32^. The receptor grid box size was 60 × 60 × 60, and the Lamarckian genetic algorithm was 1,000,000 for an individual run with root mean square at 2.0 Å. The top-ranked docked score was recorded out of 100 docking runs. After that, the binding interaction of ligand–protein was visualized by BioVia-Discovery studio software-2017^30^, and the binding interaction with ligands in terms of bond length between the functional group of the ligand and the active amino acid of the target was analyzed by protein–ligand interactions.

### Research involving plants

We confirmed that all methods (collection) involving biogenic synthesis of silver nanoparticles (AgNPs) with leaf extract of *Chromolaena odorata* (Asteraceae) in the current study strictly adhere to institutional guidelines and have been verified at the Central Research Laboratory, IMS & SUM Hospital, Siksha ‘O’ Anusandhan University, Odisha, India. The formal identification of the plant material was conducted by botanist Dr. Rabindra N. Padhy, a former HOD of the Botany Department, B. J. B. Autonomous College, Bhubaneswar, Odisha. Additionally, the voucher specimen with the number PTBG0000025473 is in the National Tropical Botanical Garden Herbarium. This voucher and its details, including taxon classification as *Chromolaena odorata* (Asteraceae) under the PIER taxon, can be verified at http://www.hear.org/vouchers/pier/ptbg0000025473.htm.

### Statistical analysis

All data were expressed as the mean ± standard deviation (S.D.). All measurements were performed by using one-way analysis of variance with Statistical Package for Social Sciences/20 software for expressing the significance of the paper. A *p*-value less than 0.05 was considered statistically significant.

### Ethical approval

This article does not contain any studies with human participants or animals performed by any of the authors. The manuscript is submitted with the consent of all authors.

## Results and discussion

The aqueous extract of dried leaf powders of *C. odorata* was used to synthesize AgNPs with the plant, and the color of the mixture-solution changed from yellowish to dark brown after 15 min of adding C. odorata leaf extract to silver nitrate solution, which was a clear indicator of the formation of AgNPs.

### Characterization

**Figure 2:**
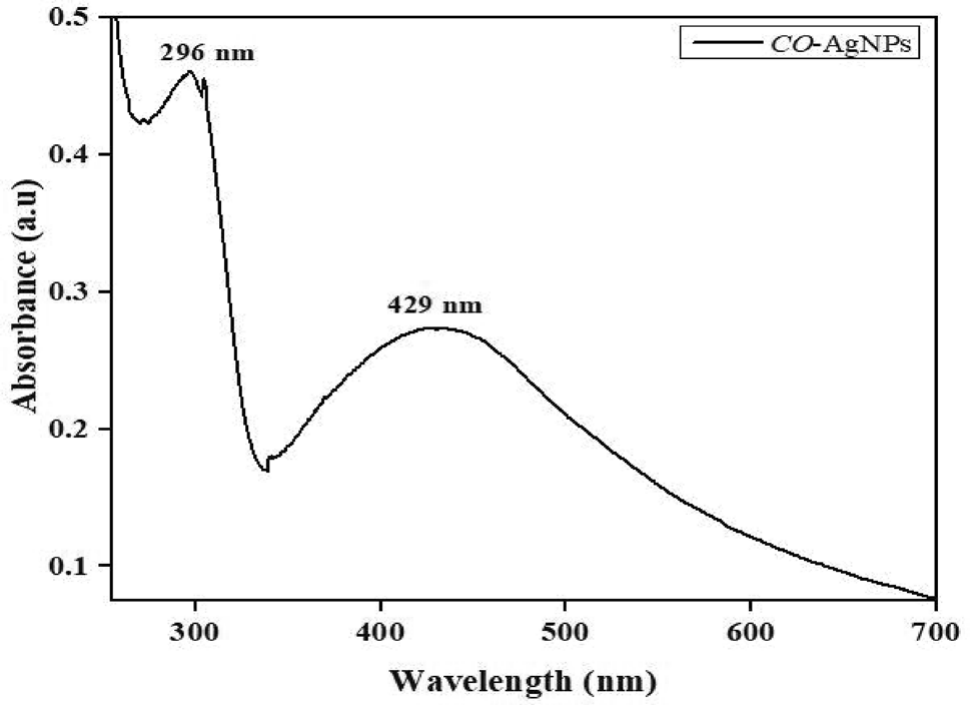
UV–Vis spectrophotometer of synthesized *CO*-AgNPs.

The UV–vis spectroscopy was used to examine the biosynthesis of *CO*-AgNPs, and the absorption in the UV–vis spectra range was 400–500 nm; thus, the green synthesis of silver nanoparticles with *C. odorata* leaf extract and their characteristic of UV–vis absorption peak shown at 450 nm^22^. AgNPs were confirmed to possess surface-free electrons that produced the surface plasmon resonance (SPR) band when interacting with an electromagnetic wave. It was revealed that the water-soluble fraction of the leaf extract played a complex/critical role by serving as the precursors for bio reduction, and the formation of due shapes of nanoparticles, as suggested^37^. The color change confirmed the UV–visible wavelength peak of the mixture lot at 429 nm, a sign that AgNPs were formed (Fig. 2).

The XRD analysis was used to determine the crystal size and shape of *CO*-AgNPs. The synthesized *CO*-AgNPs with the leaf extract of *C. odorata* were characterized by XRD patterns that typically peak (Fig. S3). An XRD investigation confirmed the crystalline structures of AgNPs and represented the main peak at (2Ө) 27.81°, 32.14°, 38.03° and 44.20°, which corresponds to plane 210, 122, 111, and 200 with half of the maximum intensity (FWHM) was 0.33°. The average particle size was 82.12 nm as calculated by Scherr’s equation^31,38^.

**Figure 3:**
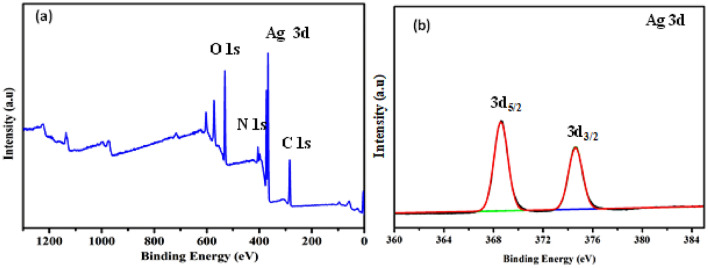
XPS spectra of silver nanoparticles (**a**) XPS of silver nanoparticles, (**b**) XPS of Ag 3d.

The XPS spectrum of the surface of silver nanoparticles is shown in Fig. 3. The elements of Ag, O, C, and N were detected. Two peaks of Ag occurred at 368.3 eV and 374.3 eV (Fig. 3a), which correspond to Ag 3d5/2 and 3d3/2 binding energies (Fig. 3b), respectively^33,34^.

The probable biomolecules responsible for the capping of AgNPs in *C. odorata* leaf extract, resulting in effective stabilization of *CO*-AgNPs, were identified by the FTIR analysis (Fig. S4). The band at 3354 cm^−1^ in the FTIR spectra of AgNPs corresponds to the OH stretching of phenolic compounds, as shown previously^39^_,_ and the vibration bands were at 2960 cm^−1^ (C–H str.), and 1580 cm^−1^ (C–C str.) were from aromatic rings from plant metabolites^40^. In this work, the peaks were at 1035 cm^−1^, 1108 cm^−1^ (C–O str.), and 780 cm^−1^ are aromatic groups. After bio-reduction, the absorption band of 1580 cm^−1^ identified the formation of AgNPs, which were capped by bio-moieties. The functional groups of plants shown by Dwivedi and Gopal^40^, interacted with the biological components through metal salts and mediated their reduction to nanoparticles.

**Figure 4:**
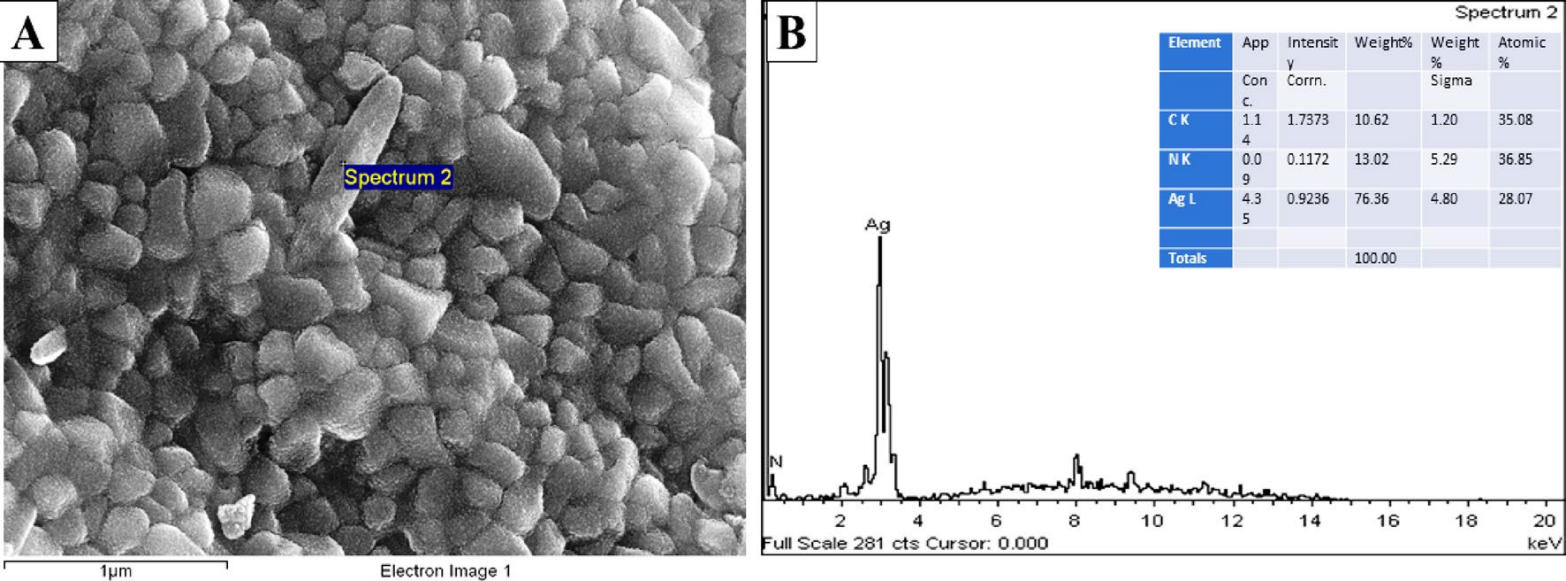
(**A**) FESEM image of synthesized of *CO*-AgNPs. (**B**) EDX spectrum of synthesized *CO*-AgNPs showing the presence of silver (Ag).

The morphological analysis of synthesized *CO*-AgNPs was carried out by FESEM analysis (Fig. 4A), which showed the spherical-shaped and poly-dispersed nature of *CO*-AgNPs^16^. The surface morphology of the solution of *CO*-AgNPs revealed the nanoparticles in irregular forms; indeed, the smaller spherical and irregularly shaped particles of various sizes were visible. The inherent chemicals of the plant could have caused the changes in particle sizes. The elemental composition of powdered materials was determined using an EDX detector. EDX study indicated a significant signal in the silver area 76.36%, confirming the synthesis of AgNPs as well as, the presence of other elemental signals such as carbon 10.62% and nitrogen 13.02% (Fig. 4B). Moreover, due to the surface plasmon resonance, AgNPs had shown a characteristic optical absorption peak of approximately three keV^38,41^.

**Figure 5:**
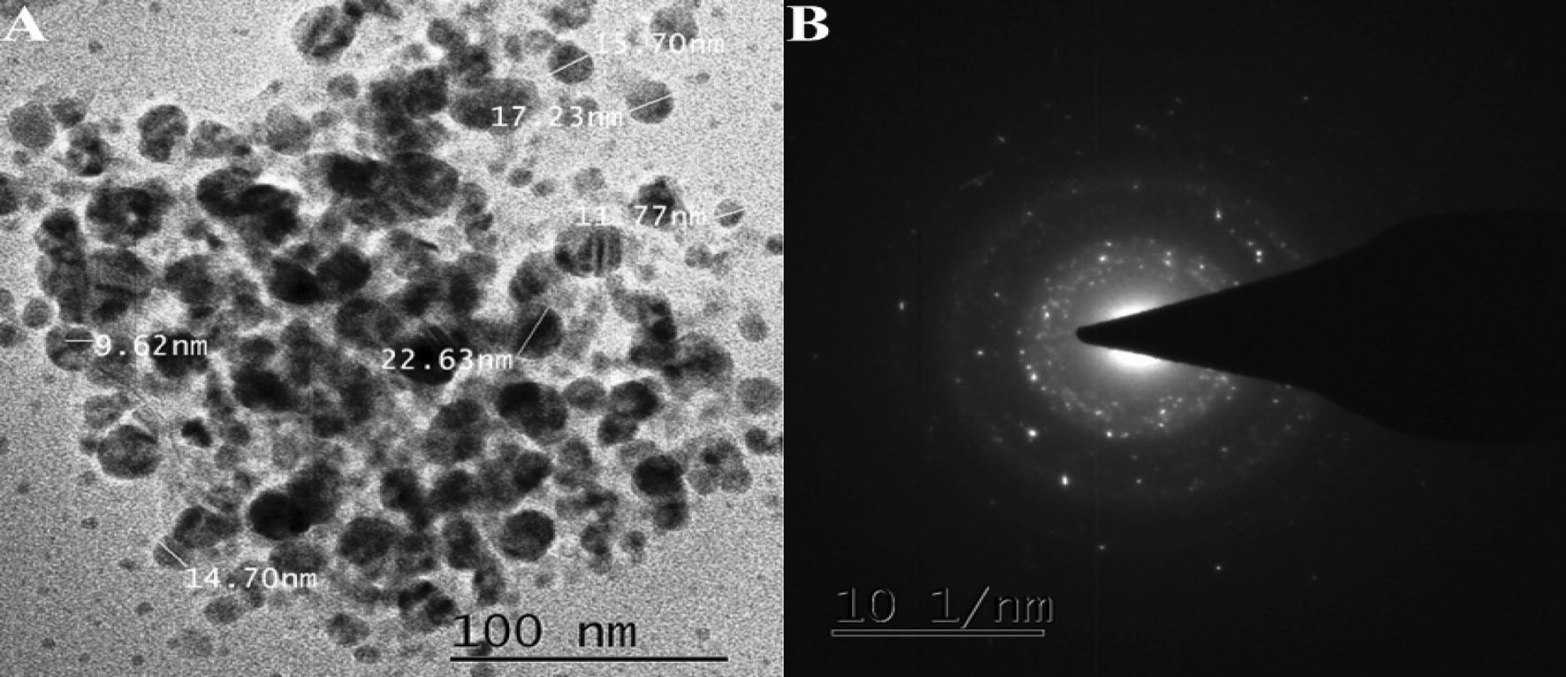
(**A**) TEM image of synthesized of *CO*-AgNPs. (**B**) The selected area electron diffraction (SAED) pattern.

TEM was used to elucidate the size and form of the resulting particles. A carbon-coated copper grid was covered with aliquots of an Ag nanoparticle solution, which were then allowed to dry naturally while TEM images were captured. According to the TEM micrographs, the size of particles had a spherical shape. Their sizes were from 9 to 22 nm with an average length of 15.27 nm (Fig. 5A) and shown the selected area electron diffraction (SAED) pattern (Fig. 5B). However, the green synthesis of silver nanoparticles was carried out using *Pedalium murex* leaf extract. Their TEM investigation revealed a spherical form with a size of 50 nm^31^.

Dynamic light scattering (DLS) was used to investigate the AgNPs generated using *C. odorata* aqueous leaf extract, and nanoparticle size distribution was mentioned. DLS evaluated the size distribution of biosynthesized *CO*-AgNPs with a size range of 24 to 37 nm and an average size of 30.5 nm (Fig. S5A) and zeta potential (ξ) − 0.532 Mv (Fig. S5B). The silver nanoparticles' negative zeta potential value could be caused by the possible capping of the bioorganic elements in the plant extracts onto the particle surface^42,43^. The smaller particle size distribution indicated that the synthesized particles were in this range.

### Biological activities

The *C. odorata* leaf powdered extract was first combined with several solvents: ethanol, methanol, butanol, diethyl ether, n-hexane, and distilled water. However, the aqueous leaf extract was used for the synthesis of AgNPs. The biosynthesized different spectra analyses characterized the sample and confirmed the synthesis of AgNPs. After that, the solvent-mixed sample and *CO-*AgNPs were used to monitor antimicrobial actions against bacterial and fungal strains on the agar well diffusion process and MIC 96-well microplates. The standard antibiotic drug, Gentamicin 10 µg/mL was applied against bacteria and Nystatin 50 µg/mL for fungi, which worked as positive controls.

The antimicrobial activity report recorded herein confirmed that the *CO-*AgNPs were more effective than the individually extracted solvent mixed samples. The solvent-mixed samples were resistant to the three pathogenic bacterial strains and two fungal strains except for the butanol solvent extract. All the solvent extract samples had *in-vitro* control activity against *K. pneumoniae* only. However, the *CO-*AgNPs sample had significant antifungal activity against fungal stains, *C. tropicalis,* and *T. rubrum,* and moderate activities against the used bacterial strains were seen. Hridhya and Kulandhaivel^44^, studied of antimicrobial activity of the ethanolic solvent extracts of *C. odorata* and showed more effectiveness.

**Figure 6:**
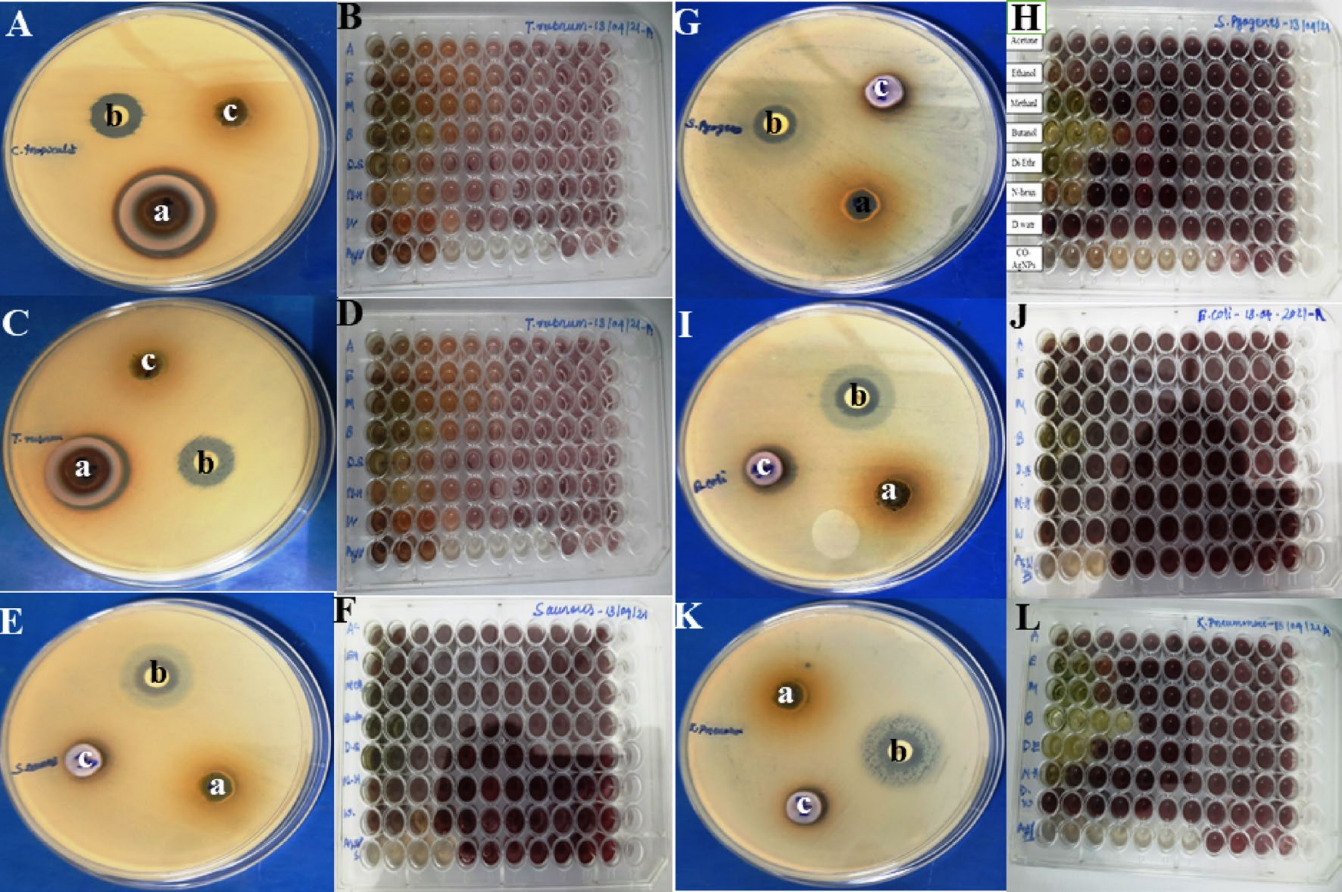
Antifungal activities (ZOI) of *CO*-AgNPs against fungi *C. tropicalis* (**A**), MIC microplate (**B**) and *T.* rubrum (**C**) and MIC microplate (**D**). Antibacterial activities (ZOI) of *CO*-AgNPs against bacteria *S. aureus* (**E**), MIC microplate (**F**); *S. pyogenes* (**G**), MIC microplate (**H**); *E. coli* (**I**), MIC microplate (**J**) and *K. pneumoniae* (**K**), MIC microplate assay (**L**). The agar well diffusion assay was indicated well as *CO*-AgNPs (a), Positive control (b) and Negative control (c). The MIC microplates were indicated well as solvent plant extract, Acetone (1), Ethanol (2), Methanol (3), Butanol (4), Diethyl ether (5), n-Hexane (6), Distilled water (7) and synthesis *CO*-AgNPs (8).

**Figure 7:**
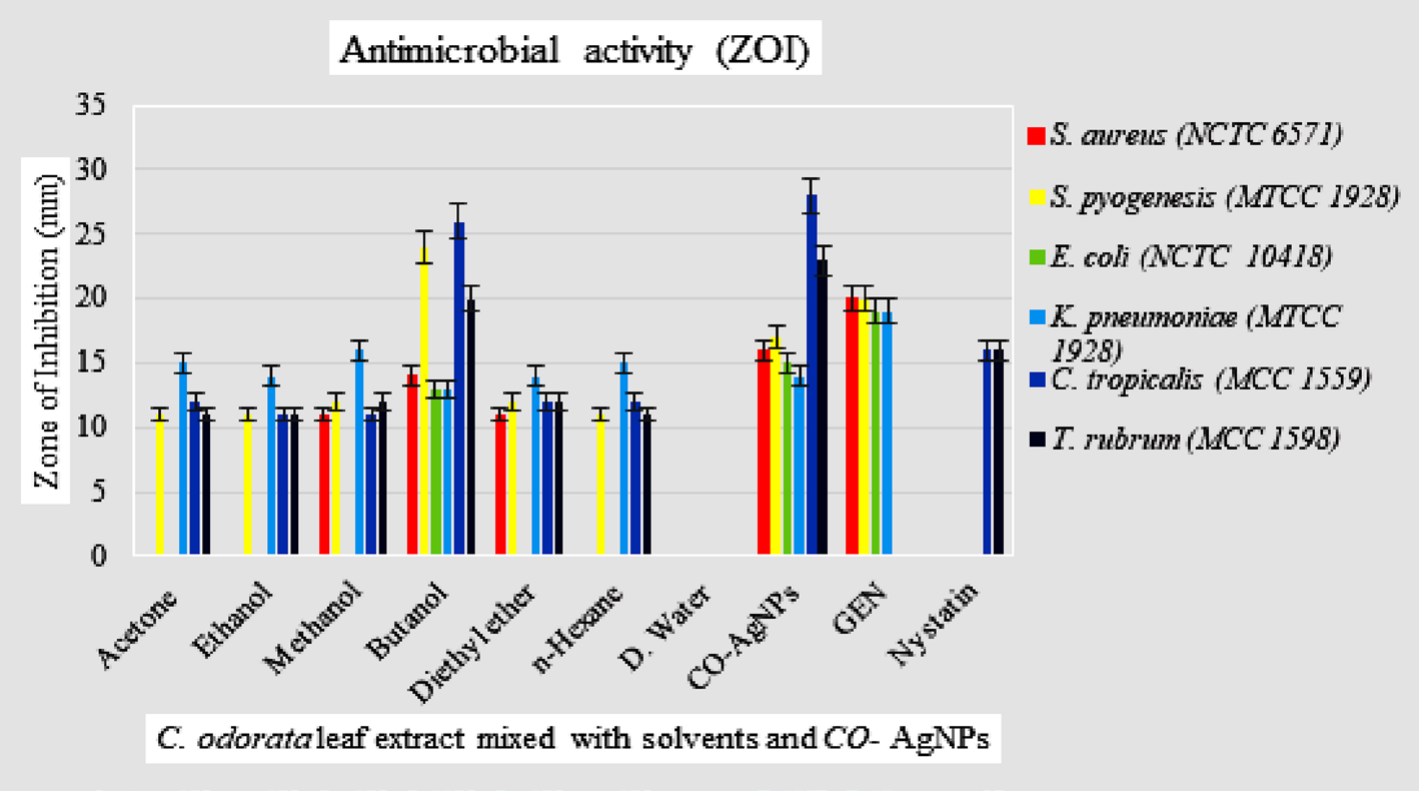
Histogram of antimicrobial activity of solvent extract and *CO*-AgNPs.

For the antimicrobial assay, the two Gram-positive bacteria, *S. aureus* and *S. pyogenes,* and two Gram-negative bacteria, *E. coli,* and *K. pneumoniae,* and the antifungal study of the two fungal strains, *C. tropicalis* and *T. rubrum* were used. The highest zones of inhibition against fungi *C. tropicalis* were 28 mm with a MIC value of 0.195 mg/mL (Fig. 6A,B); *T. rubrum* had a zone of inhibition of 23 mm with a MIC value of 0.390 mg/mL (Fig. 6C,D). The zone of inhibition was against bacterial strains, *S. aureus* at 15 mm and MIC value of 3.125 mg/mL (Fig. 6E,F); for *S. pyogenes* the zone of inhibition was at 16 mm and MIC value was 0.781 mg/mL (Fig. 6G,H); for *E. coli* it was at 14 mm and MIC value was 12.5 mg/mL (Fig. 6I,J); for *K. pneumoniae* it was at 13 mm and the MIC value was 0.781 mg/mL (Fig. 6K,L). Thus, the zone of inhibition and MIC of the solvents mixed *C. odorata* sample and *CO*-AgNPs were summarized (Table 1). Thus, the interpretations of the results are illustrated in the bar graph (Fig. 7).

**Table 1:** Antimicrobial activity of solvent extracts of *C. odorata* and *CO*-AgNPs against human pathogenic microorganisms.

Antimicrobial Activity (ZOI)												
Microorganisms** → **	*S. aureus*		*S. pyogenes*		*E. coli*		*K. pneumoniae*		*C. tropicalis*		*T. rubrum*	
Solvent extracts	ZOI* (mm)	MIC (mg/ml)	ZOI* (mm)	MIC (mg/ml)	ZOI* (mm)	MIC (mg/ml)	ZOI* (mm)	MIC (mg/ml)	ZOI* (mm)	MIC (mg/ml)	ZOI* (mm)	MIC (mg/ml)
Acetone	NA	NA	10.16 ± 1.25	50	NA	NA	14.5 ± 0.5	50	12.16 ± 1.25	12.5	10.5 ± 0.5	25
Ethanol	NA	NA	10.5 ± 1.32	50	NA	NA	13.16 ± 1.04	25	10.83 ± 0.76	12.5	10.83 ± 0.28	12.5
Methanol	10.66 ± 1.52	50	12.16 ± 0.76	25	NA	NA	15.5 ± 0.5	25	11.5 ± 0.5	12.5	11.5 ± 0.5	12.5
Butanol	14.16 ± 1.25	25	21.83 ± 1.25	12.5	13.5 ± 0.5	25	13.33 ± 1.25	6.25	24.16 ± 0.76	6.25	19.83 ± 0.76	6.25
Diethyl ether	12.16 ± 1.04	25	11.83 ± 0.76	25	NA	NA	13.83 ± 0.76	25	12.5 ± 0.5	50	11.16 ± 0.76	25
n-Hexane	NA	NA	10.5 ± 0.5	25	NA	NA	14.16 ± 1.04	50	12.66 ± 0.76	12.5	10.83 ± 0.76	25
D. Water	NA	NA	NA	NA	NA	NA	NA	NA	NA	NA	NA	NA
*CO-*AgNPs	16.83 ± 1.75	3.12	18.5 ± 0.5	0.78	15.66 ± 0.76	12.5	13.16 ± 1.25	0.78	26.83 ± 0.28	0.19	23.16 ± 0.76	0.39
GEN	21.33 ± 1.52	50 (µg/mL)	20.5 ± 0.86	50	20.16 ± 0.76	≤ 8.0 (µg/mL)	19.16 ± 0.76	50	–	–	–	–
Nystatin	–	–	–	–	–	–	–	–	17.16 ± 1.25	3.12 (µg/mL)	16.83 ± 0.76	3.12 (µg/mL)

ZOI, Zone of Inhibition; MIC, Minimum Inhibition Concentration; NA, Not available; *S. aureus*, *Staphylococcus aureus*; *S. Pyogenes*, *Streptococcus pyogenes*; *E. coli*, *Escherichia coli*; *K. pneumonia*, *Klebsiella pneumoniae*; *C. tropicalis*, *Candida tropicalis*; *T. rubrum*,* Trichophyton rubrum*; GEN, Gentamicin.

*ZOI data were calculated the Mean ± Standard deviation (SD).

### Molecular docking

**Figure 8:**
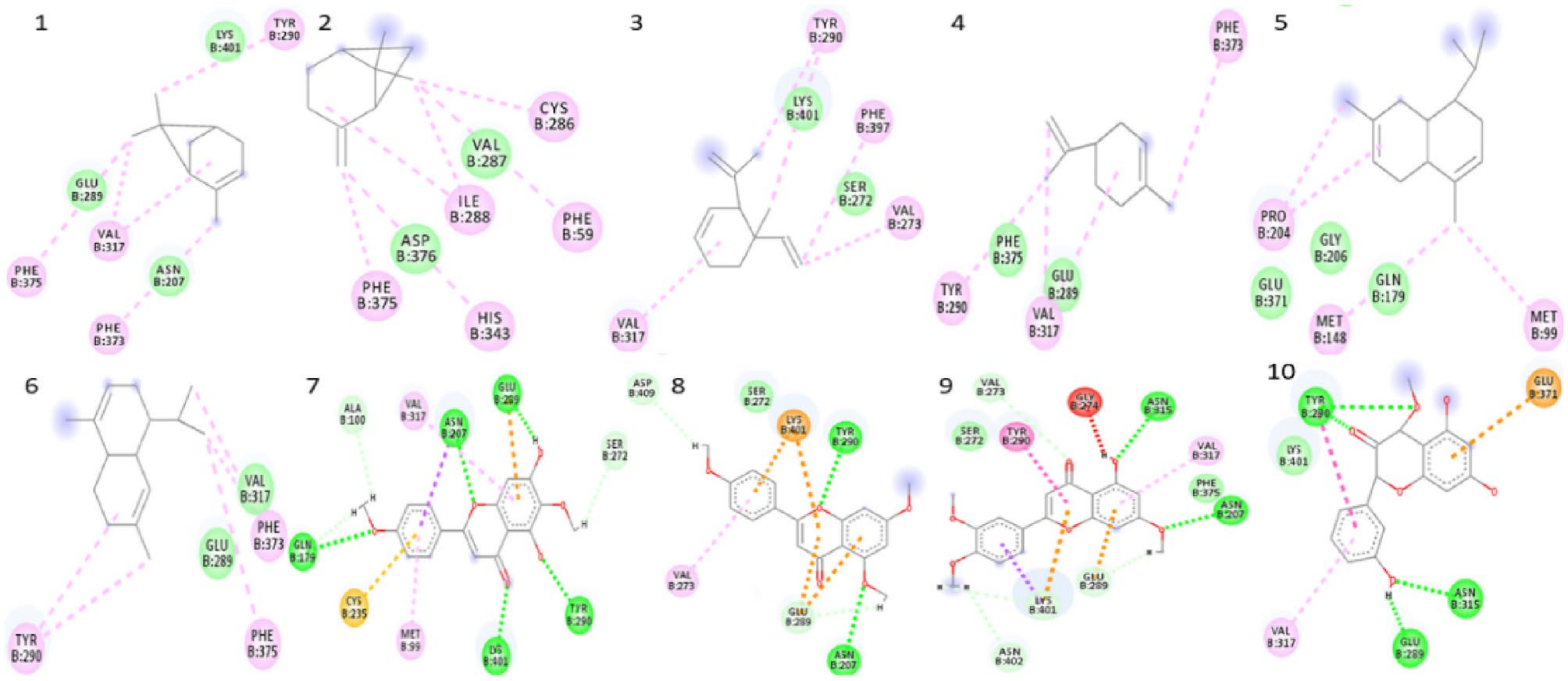
2D ligand interactions structure of the bioactive compounds 1–10 with *C. albicans* fungal target protein 5V5Z visualized by BIOVIA Discovery Studio*.*

Preliminary investigations for all probable binding affinity with the targeted enzyme and their interactions with the bioactive compounds of *C. odorata* predicted their theoretical biological data of ten bioactive compounds. The molecular docking study was performed against the bacterial protein dihydrofolate synthetase, PDB ID: 1AD1, 1AJ0, and fungal protein Lanosterol C14 α-Demethylase protein, PDB ID: 5V5Z with bioactive compounds of *C. odorata* (1–10). The molecular docking scores and binding interactions of bioactive compounds with protein were shown (Table 2). The ligand–protein interactions of potent bioactive compounds of *C. odorata* against the bacterial proteins 1AD1 and 1AJ0 (Figs. S6, S7) and fungal protein 5V5Z (Fig. 8) were visualized by BIOVIA Discovery Studio.

**Table 2:** Molecular docking and binding interaction of the bioactive compounds of *C. odorata* against bacterial and fungal targeted proteins.

Sl. no	Bioactive compounds	Chemical structure	Activity	Docking Score and binding interaction					
				*S. aureus* (PDB ID- 1AD1)		*E. coli* (PDB ID- 1AJ0)		*C. albicans* (PDBID- 5V5Z)	
				Docking Score (Kcal/mol)	Binding interaction	Docking Score (Kcal/mol)	Binding interaction	Docking Score (Kcal/mol)	Binding interaction
	IUPAC Name								
1	α-Pinene^a^		Antimicrobial activity against *B. cereus* and *A. niger*	− 5.3	Lys 159, Ile 8, His 228	− 5.4	Lys 221, Arg 63, Phe 190, Pro 64	− 5.8	Tyr 290, Phe 373, Val 317, Phe 375
	2,6,6-trimethylbicyclo[3.1.1]hept-2-ene								
2	β-Pinene^a^		Antimicrobial activity against *B. cereus* and *A. niger*	− 5.5	His 228, Phe 159	− 5.3	Lys 221, Pro 64, Arg 63, Phe 190	− 5.3	Cys 286, Phe 59, Ile 288, His 343, Phe 375
	6,6-dimethyl-2-methylidenebicyclo[3.1.1]heptane								
3	Geijerene^a^		Antimicrobial activity against *B. cereus* and *A. niger*	− 5.5	His 228, Ile 8, Phe 159	− 5.6	Arg 63, Lys 221, Ile 20, H257	− 5.6	Val 317, Tyr 290, Val 273, Phe 397
	4-ethenyl-4-methyl-3-prop-1-en-2-ylcyclohexene								
4	Limonene^a^		Antimicrobial activity against *B. cereus* and *A. niger*	− 5.1	Ile 8, His 228, Met 115, Phe 159	− 5.1	Phe 190, Lys 221, Arg 63	− 5.8	Phe 373, Val 317, Tyr 290
	1-methyl-4-prop-1-en-2-ylcyclohexene								
5	β-Cadinene^a^		Antimicrobial activity against *B. cereus* and *A. niger*	− 6.3	His 42, Lys 190, His 228, Phe 159	− 6.2	Lys 221, Arg 63, Phe 190	− 6.0	Met 148, Pro 204, Met 99
	(1*S*,4*aR*,8*aS*)-4,7-dimethyl-1-propan-2-yl-1,2,4*a*,5,8,8*a*-hexahydronaphthalene								
6	α-Cadinene^a^		Antimicrobial activity against *B. cereus* and *A. niger*	− 6.1	His 228, Ile 8, Phe 159, His 42	− 6.0	Phe 190, Arg 63, Arg 255, Lys 221, Pro 64	− 6.3	Val 317, Phe 375, Tyr 290
	(1*S*,4*aR*,8*aS*)-4,7-dimethyl-1-propan-2-yl-1,2,4*a*,5,6,8*a*-hexahydronaphthalene								
7	Pctolinaringenin^b^		Antimicrobial activity against *E. coli*, *S. aureus, K. pneumoniae*, *A. fumigatus* and *C. neoformans*	− **8.2**	Asp 71, Arg 206, Arg 191, Lys 190, Arg 39, Arg 226	− **8.3**	Thr 62, Lys 221, Arg 63	− **8.7**	Ala 100, Gln 179, Cys 235, Met 99, Lys 401, Tyr 290, Ser 272, Glu 289, Met 207, Val 317
	5,7-dihydroxy-6-methoxy-2-(4-methoxyphenyl)chromen-4-one								
8	±)-4′,5,7-trimethoxy flavanone^b^ 5,7-dimethoxy-2-(4-methoxyphenyl)chromen-4-one		Anti-biofilm and metabolic inhibition activities against *E. coli* and *S. aureus*	− 7.4	Arg 189, Ser 188, Lys 190, Arg 39, Asn 10, Arg 226	− 7.7	S222, Lys 221, Arg 255, Glu 60, Asp 96, Asn 22, Arg 63	− **8.3**	Asp 409, Lys 401, Tyr 290, Asn 207, Glu 289, Val 273
9	5-hydroxy-3,7,4′-trimethoxyflavone^b^ 2-(3,4-dimethoxyphenyl)-5-hydroxy-7-methoxychromen-4-one		Anti-biofilm and metabolic inhibition activities against *E. coli* and *S. aureus*	7.6	Arg 39, Ser 37, Gln 92, Phe 159, Arg 226, Asp 71, Lys 190	− 7.8	Arg 63, Pro 145, Asn 144, Pro 64, Thr 62, Phe 190, Lys 221	− **8.2**	Val 273, Tyr 290, Gly 274, Asn 315, Val 317, Asn 207, Glu 289, Lys 401, Asn 402
10	3,5,7-trihydroxy-4′-methoxyflavone^b^		Antimicrobial activity against *E. coli, S. aureus, K. pneumoniae*, *A. fumigatus* and *C. neoformans*	7.5	Val 36, Arg 39, Asp 71, Asn 10, Gly 34	− **8.1**	Asn 144, Thr 147, Arg 63, Phe 190, Pro 64, Ser 222, Gly 189	− **8.4**	Tyr 290, Glu 371, Asn 315, Glu 289, Val 317
	5,7-dihydroxy-2-(3-hydroxyphenyl)-4-methoxy-4*H*-chromen-3-one								

^a^Owolabi et al.^23^.

^b^ Omokhua-Uyi et al.^24^.

IUPAC- International Union of Pure and Applied Chemistry, PDB- Protein Data Bank, *S. aureus: Staphylococcus aureus, E. coli: Escherichia coli, C. albicans- Candida albicans, B. cereus*- *Bacillus cereus, A. niger- Aspergillus niger, K. pneumonia: Klebsiella pneumoniae, A. fumigatus- Aspergillus fumigatus, C. neoformans- Cryptococcus neoformans.*

Significant values are in bold.

**Figure 9:**
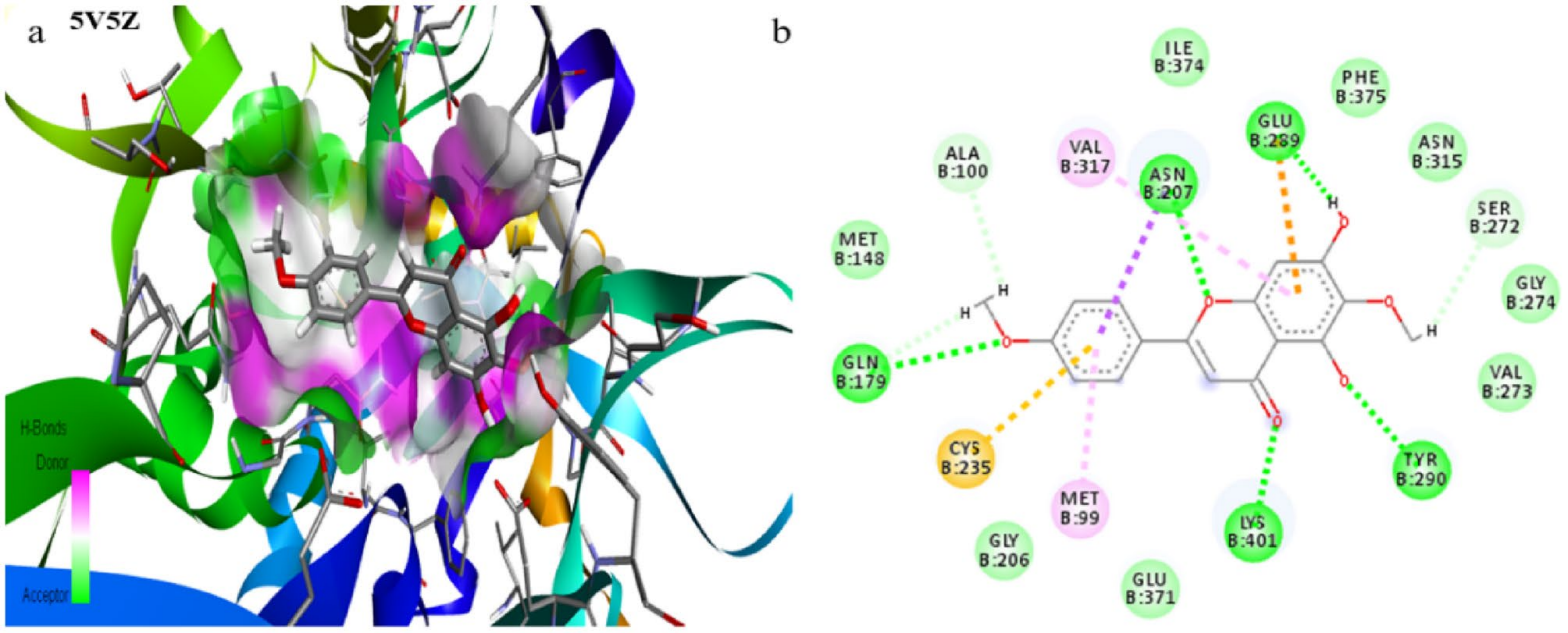
Docking and interactive bonding mode pose (**a**) and 2D ligand interaction structure (**b**) of the compound Pctolinaringenin (**7**) with fungal (*C. albicans*) target protein 5V5Z visualized by BIOVIA Discovery Studio.

Among all the phytocompounds, the Pctolinaringenin had the highest docking score of -8.7 kCal/mol, against the fungal target 5V5Z, which interacts with Ala 100, Gln 179, Cys 235, Met 99, Lys 401, Tyr 290, Ser 272, Glu 289, Met 207 and Val 317 of the amino acid residues of the target with ligand by hydrogen bonding (Fig. 9a,b).

**Figure 10:**
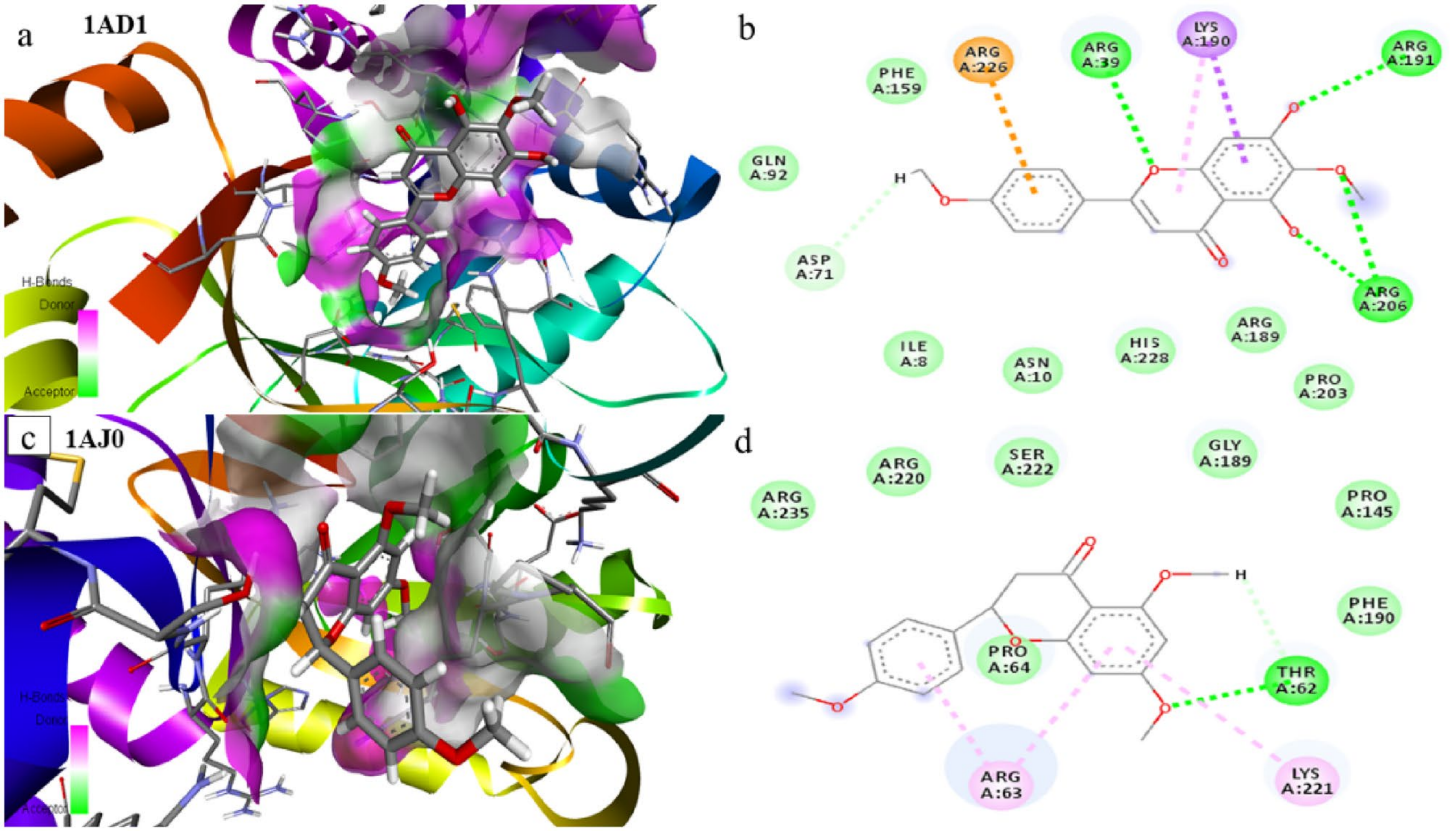
Docking and amino acid interactions (**a**), 2D ligand interaction structure (**b**), docking and interactive bonding mode pose (**c**), and 2D ligand interaction structure (**d**), of the compound Pctolinaringenin (**7**), with *S. aureus* bacterial target protein 1AD1 and *E. coli* bacterial target protein 1AJ0, visualized by BIOVIA Discovery, respectively.

The bioactive compound, Pctolinaringenin of the highest docking had scores of − 8.2, and − 8.3 against both bacterial targets 1AD1 and 1AJ0, respectively, from which the bacterial target 1AD1 interacts with Asp 71, Arg 206, Arg 191, Lys 190, Arg 39 and Arg 226 of the target amino acid residue by H-bonding (Fig. 10a,b). Furthermore, the other bacterial target, 1AJ0 interacts with Thr 62, Lys 221, and Arg 63 of the target's amino acid residues by H-bond (Fig. 10c,d).

Indeed, *C. odorata* is a rich source of novel bioactive secondary metabolites such as Limonene, Odoratenin, Subscandenin, Dihydrokaempferide, Isosakuranetin, Kaempferide, Acacetin, and Cinnamaldehyde, which have been identified earlier as potential lead compounds for the development of antimicrobial, antioxidant, and anticancer agents^23,45, 46^. Therefore, the current work of *CO*-AgNPs represents the drug-development effort, with bioinformatics principles assisting in designing chemicals to identify suitable hybrid chemicals as potential future antibacterial and antifungal agents.

## Conclusion

In the present study, biosynthesized *CO-*AgNP samples showed an absorbance peak at 427 nm in UV–vis spectroscopy*,* indicating that the AgNPs were biosynthesized. Analyses with UV–vis spectroscopy, DLS and zeta potential, and FESEM-EDX confirmed the presence of AgNPs. These analyses suggested three major distributions of particle sizes. Moreover, the TEM analysis of biosynthesized AgNPs with a size range of 9–22 nm with an average size of 15.27 nm; and the obtained X-ray diffraction peak of 38.03° was obtained to confirm the crystalline structure of the *CO*-AgNPs. Furthermore, the biosynthesized *CO*-AgNPs displayed strong antifungal efficacy against fungi *C. tropicalis* and *T. rubrum*, and some bacterial species. The molecular docking study further revealed that the compound Pctolinaringenin would be a plausible therapeutic candidate against microbial diseases. Thus, the biological synthesis method of AgNPs exhibited several advantages and in vitro applications. In the future, it could be targeted for formulating various agents and other biological applications in particular against fungal-associated diseases.

### Supplementary Information

Supplementary Figures.

## Data Availability

The datasets generated during and/or analyzed during the current study are available from the corresponding author on reasonable request.
