# Supplementary material for: Unveiling the antibacterial and antifungal potential of biosynthesized silver nanoparticles from Chromolaena odorata leaves
Source: Sci Rep. 2024 Mar 29;14:7513. doi: 10.1038/s41598-024-57972-5 (PMC10980689; doi:10.1038/s41598-024-57972-5)
Supplement: Supplementary file 1 — Supplementary Figures. [file 41598_2024_57972_MOESM1_ESM.doc]

**Unveiling the Antibacterial and antifungal potential of biosynthesized silver nanoparticles from *Chromolaena* *odorata* leaves**

**Ajit Kumar Bishoyi^a,b^, Chita Ranjan Sahoo^a^, Priyanka Samal^b^, Nilima Priyadarsini Mishra^e^, Bigyan Ranjan Jali^c*^, Mohd Shahnawaz Khan^d*^, Rabindra Nath Padhy^a*^**

^a^ Central Research Laboratory, Institute of Medical Sciences & Sum Hospital, Siksha ‘O’ Anusandhan Deemed to Be University, Bhubaneswar, Odisha, 751003, India

^b^ Department of Clinical Hematology, Institute of Medical Sciences & Sum Hospital, Siksha ‘O’ Anusandhan Deemed to Be University, Bhubaneswar, Odisha, 751003, India

^c^Department of Chemistry, Veer Surendra Sai University of Technology Burla Sambalpur Odisha 768018, India

^d^Department of Biochemistry, College of Science, King Saud Unoversity, Riyadh, 11451, Saudi Arabia

^e^Post Doctoral fellow, Department of Biochemistry, IIT Ropar 140001, Punjab India

*Corresponding authors: E-mail: bigyan.Jali7@gmail.com (Dr. Jali), [rnpadhy54@gmail.com](mailto:rnpadhy54@gmail.com) (Prof. Padhy) [moskhan@ksu.edu.sa](mailto:moskhan@ksu.edu.sa) (Dr. Khan)

**Supplementary Materials**

**Unveiling the Antibacterial and antifungal potential of biosynthesized silver nanoparticles from *Chromolaena* *odorata* leaves**


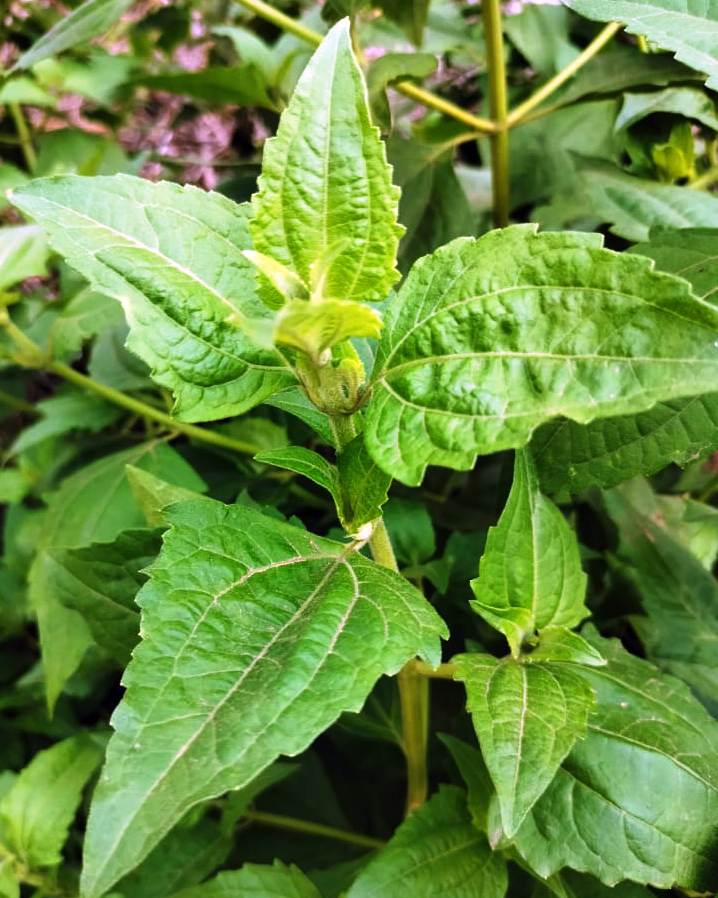


**Fig. S1** Plant *Chromolaena odorata*.


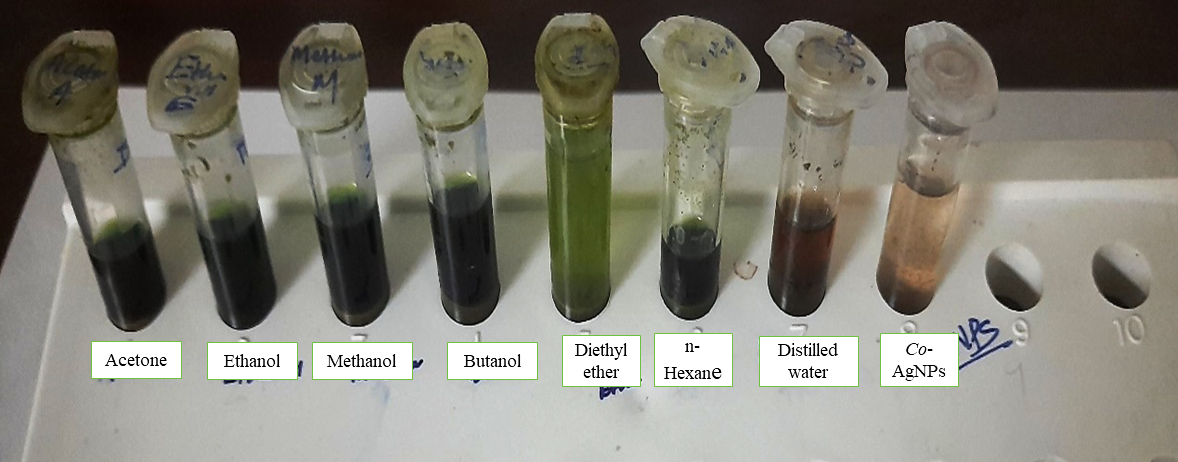


**Fig. S2** *C. odorata* leaf powder extract mixed with different chemical solvents.


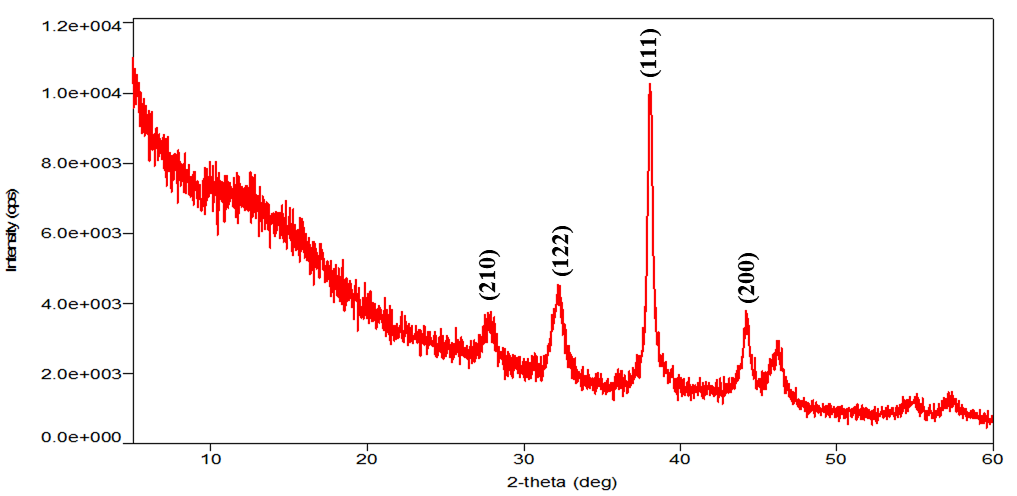


**Fig. S3** XRD spectra pattern of synthesized of *CO*-AgNPs.


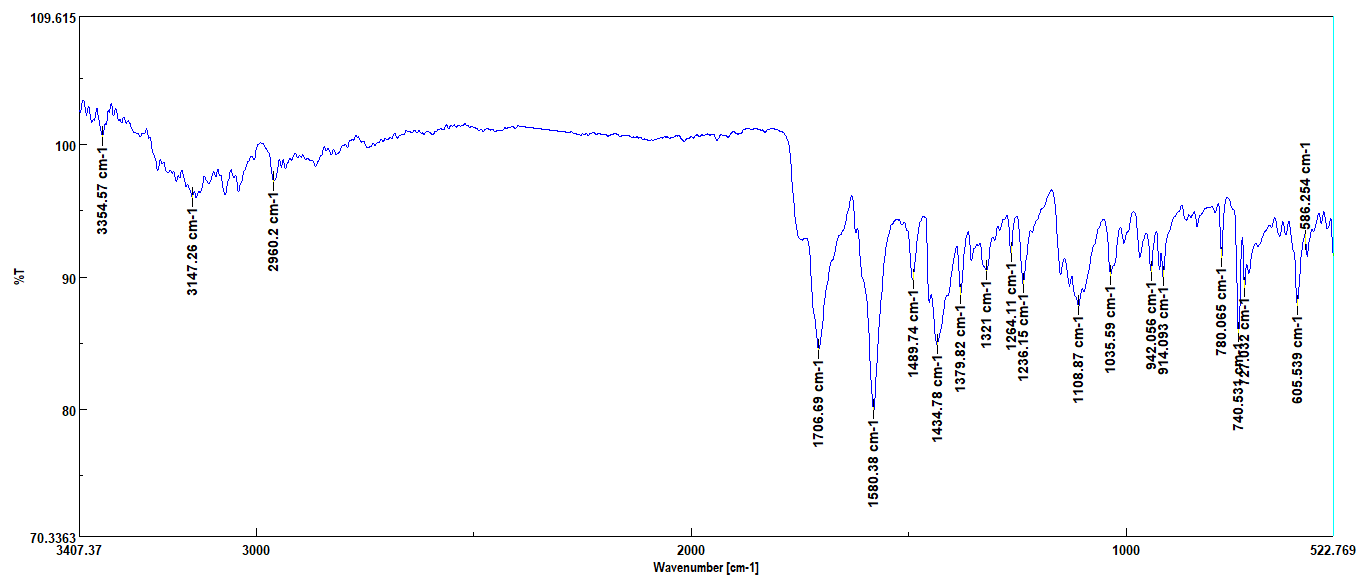


**Fig. S4** FTIR spectra of synthesized of *CO*-AgNPs.


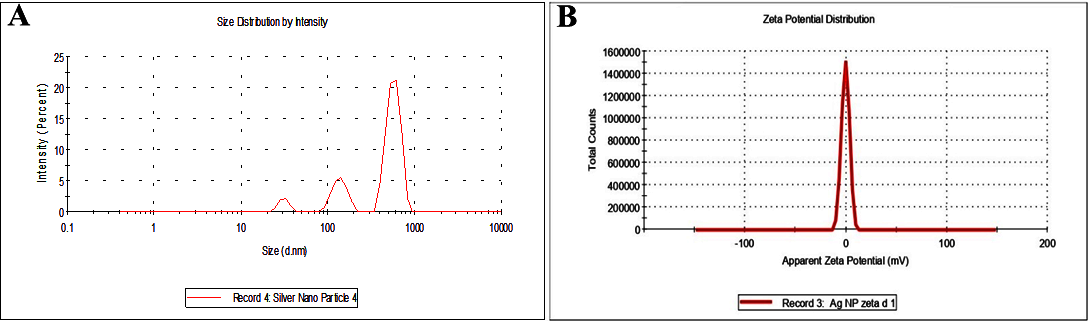


**Fig. S5** (A) DLS size distribution by intensity and (B) zeta potential distribution of synthesized *CO*-AgNPs.


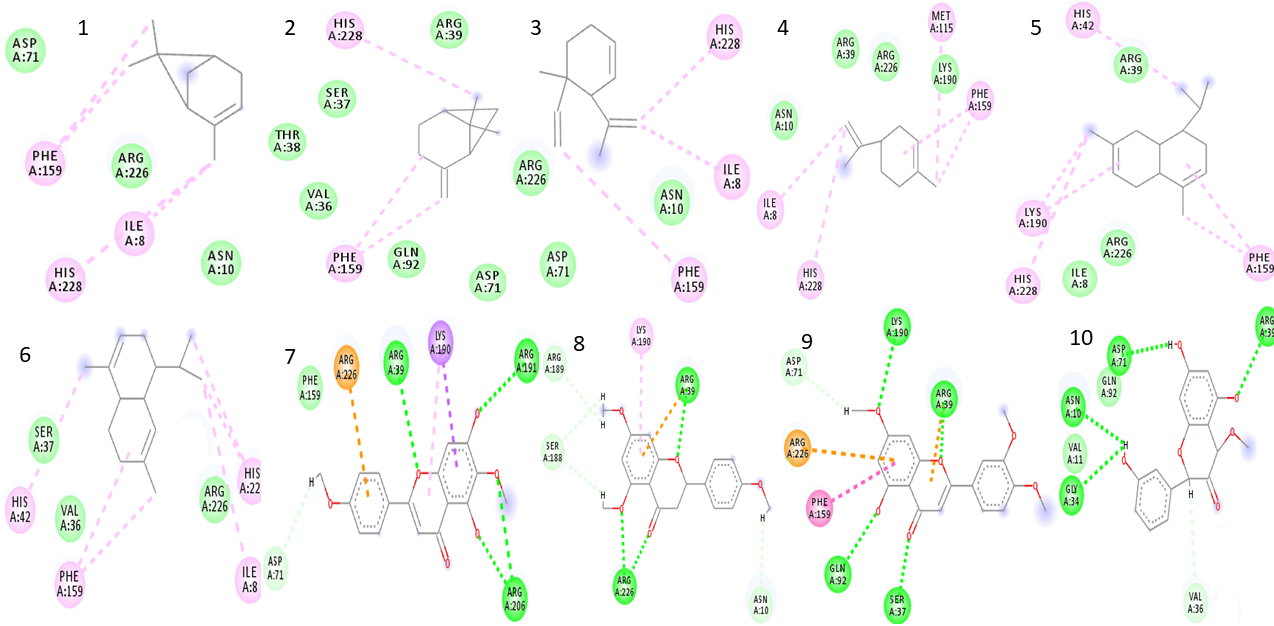


**Fig. S6** 2D ligand interactions structure of the bioactive compounds 1-10 with *S. aureus* bacterial target protein 1AD1 visualized by BIOVIA Discovery Studio*.*


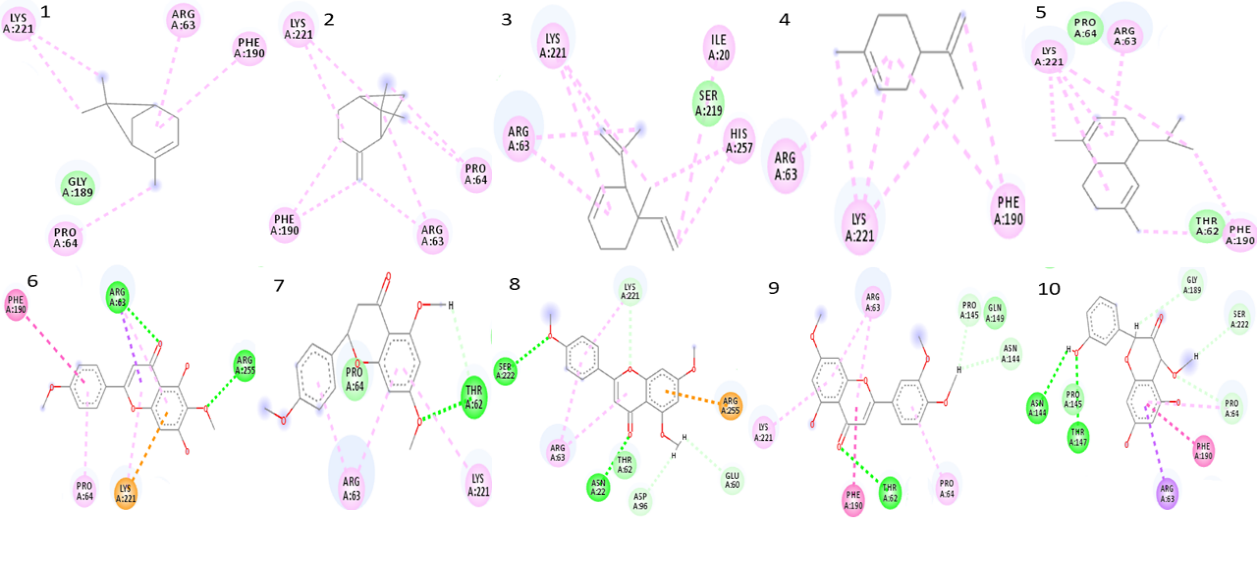


**Fig. S7** 2D ligand interactions structure of the bioactive compounds 1-10 with *E. coli* bacterial target protein 1AJ0 visualized by BIOVIA Discovery Studio*.*
